# A concomitant of subarachnoid hemorrhage and idiopathic renal rupture in a hemodialysis patient with COVID-19

**DOI:** 10.12669/pjms.41.5.10718

**Published:** 2025-05

**Authors:** Yanan Zhang, Hongjian Wei, Ruojing Wei

**Affiliations:** 1 Yanan Zhang 2nd Department of Urology, Baoding No.1 Central Hospital of Hebei Medical University, Baoding 071000, Hebei, China; 2 Hongjian Wei 2nd Department of Urology, Baoding No.1 Central Hospital of Hebei Medical University, Baoding 071000, Hebei, China; 3 Ruojing Wei 2nd Department of Urology, Baoding No.1 Central Hospital of Hebei Medical University, Baoding 071000, Hebei, China

**Keywords:** Hemodialysis, Renal rupture, Subarachnoid hemorrhage, Superselective renal artery embolization

## Abstract

Idiopathic renal rupture (IRR) is considered an emergency condition in the field of urology. While some cases may have unknown causes, the majority are attributed to specific underlying conditions such as kidney tumors, inflammation, stones, vascular diseases, and coagulation disorders. It is important to note that IRR is not commonly observed in patients undergoing hemodialysis. Herein, we present a unique case of a middle-aged male patient with chronic nephritis syndrome and COVID-19.

The individual experienced severe headache and abdominal pain during the initial stages of hemodialysis, leading to the timely diagnosis of subarachnoid hemorrhage (SH) and IRR. Following the diagnosis, the patient underwent symptomatic conservative treatment for SH and super-selective renal artery embolization for IRR, resulting in gradual relief of his symptoms within three days. After a week, the patient’s overall condition showed significant improvement. To the best of our knowledge, this is the first documented instance of a hemodialysis patient with COVID-19 experiencing SH concurrent with IRR.

## INTRODUCTION

Idiopathic renal rupture (IRR), also known as Wunderlich syndrome, refers to the occurrence of renal parenchyma and pelvis, or blood vessel rupture in the absence of any trauma. This leads to acute spontaneous hemorrhage in the renal capsule, peri-renal area, and retroperitoneum. Clinically, it is often characterized by acute abdominal or flank pain, abdominal mass, and the triad of bleeding. This disease is relatively rare and progresses rapidly. If not diagnosed and treated promptly, it can lead to hemorrhagic shock and even be life-threatening. Subarachnoid hemorrhage (SH) is a common cerebrovascular disease among patients undergoing hemodialysis and is also one of the common causes of death in such individuals. Herein, we present a case report on a COVID-19 patient undergoing hemodialysis, who also experienced concurrent SH and IRR. To the best of our knowledge, there are currently no clinical reports on such patients.

## CASE PRESENTATION

**Fig.1:**
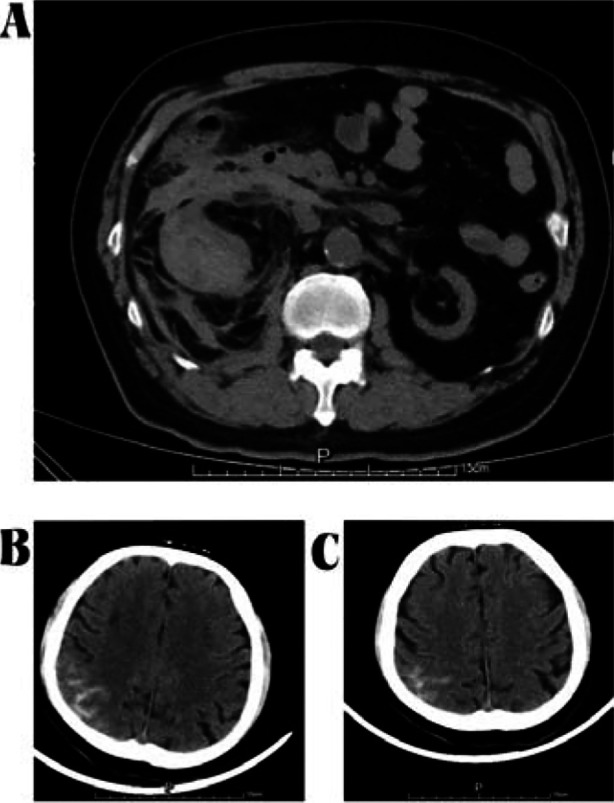
Computed tomographyscan of the abdomen showed abnormal morphology, disappeared renal portal structure, strip slightly high density shadow, surrounding multiple flocculous high density and unclear boundary in the right kidney and indicated him renal rupture (A) Computed tomographyscan of the skull showed high-density shadow in the top part of the right brain groove and indicated him subarachnoid hemorrhage at admission (B) and Three days after admission (C).

**Fig.2:**
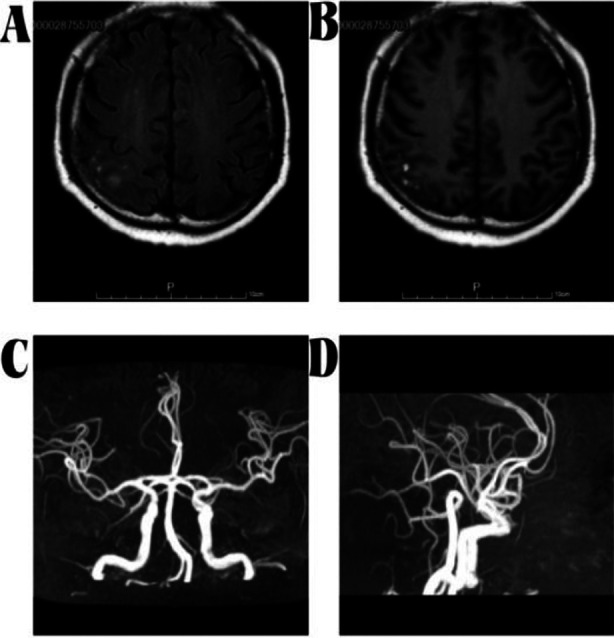
Magnetic resonance imaging of brain showed a short T1 signal shadow in the upper right sulcus of (A, B). Magnetic resonance angiography of brain showed no obvious abnormalities (C, D).

**Fig.3:**
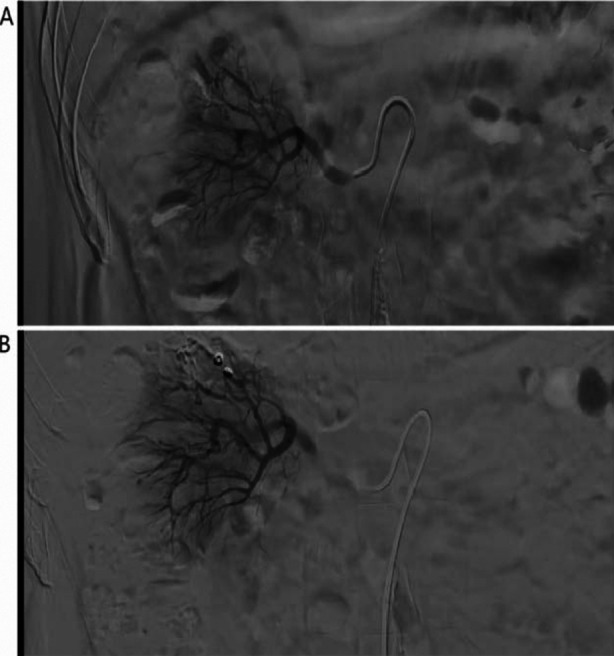
Right renal angiography showed contrast medium spilled out near the right upper renal branch artery (A) Right renal angiography showed the hemorrhage was disappeared and the injured artery and related site of kidney were occluded completely after embolization (B).

A 54 years old man with chronic nephritis syndrome who complained of severe headache and abdominal pain for one daywas admitted to our hospital. The patient was diagnosed with uremia and started hemodialysis one month ago. He was diagnosed with COVID-19 via nasopharyngeal PCR assay one week ago. He experienced persistent colic pain on the right side and near the umbilical area. His headache was durative, but he was conscious without disorder of limb’s activity. Additionally, the patient experienced nausea and vomiting. Physical examination at admission revealed right periumbilical tenderness and renal percussive pain. His heart rate and blood pressure were both normal. Emergency computed tomography (CT) scan of the skull and abdomen showed him IRR (Fig.1A) and SH (Fig.1B). Further examination with cranial magnetic resonance imaging confirmed the diagnosis of SH in the patient (Fig.2A, B) and ruled out the possibility of cerebral vascular malformations (Fig.2C, D). Subsequently, in view of the findings by neurologist and interventional radiologist examinations, the patient received symptomatic conservative treatment for SH and super selective renal artery embolization (SRAE) for IRR, respectively. The patient received conservative treatment, including blood pressure control, pain management, prevention of cerebral vasospasm (using nimodipine), and prevention of seizures (using antiepileptic drugs). Additionally, the patient underwent close neurological monitoring and supportive care. SRAE was conducted by a digital subtraction angiographic unit via the right femoral access by a Five French (Fr.) catheter. A micro catheter was used for super-selective catheterization in the right renal artery and hemorrage site near the right inferior renal branch artery was found (Fig.3A). When the injury site was discovered, the bleeding artery to the injury site was placed with a 3Fr catheter and two steel coils were used for embolizing the lesions. Renal angiography was conducted for confirming the whole occlusion in the injury vascular and ended the entire operation (Fig.3B). No complications were noted in the whole process. Through active targeted treatment, his headache and abdominal pain gradually eased three days later. Two week later, his condition improved significantly. The specific results of laboratory tests during the patient’s hospitalization can be found in Table-I. The patient underwent regular hemodialysis and his condition was stable after one month of follow-up.

**Table-I:** Laboratory examination in our hospital.

Investigation	Normal Range	Day 1	Day 3	Day 7	Day 14
Hb (g/L)	110-150	78.5	68.1	76.3	82.4
Plt (×10^9^/L)	125-350	453	331	226	345
Alb (g/L)	40-55	32.4	28.7	31.4	35.7
UREA (mmol/L)	2.60-7.50	31.4	29.7	21.4	23.5
CREA (umol/L)	41.0-73.0	884.6	774.6	476.1	542.3
Serum sodium (mmol/L)	137-145	131.4	135.3	136.4	138.2
Serum potassium (mmol/L)	3.50-5.30	4.89	5.13	4.62	4.19
Serum calcium (mmol/L)	2.11-2.52	1.96	1.87	2.12	2.14
Serum phosphorus (mmol/L)	0.85-1.51	1.86	1.78	1.53	1.27
BNP (pg/ml)	0-100	7845.6	6743.2	4845.3	2789.3
D-Dimer (mg/L)	0-0.55	1.96	1.76	1.45	1.12
CRP (mg/L)	0-10	12.3	15.1	11.4	10.7
Fbg (g/L)	1.8-3.5	3.79	4.13	3.56	2.96
LVEF (%)	45-55	48	/	/	/

*Note:* Hb (hemoglobin), Plt (platelet), Alb (albumin), CREA (creatinine), BNP (brain natriuretic peptide), Fbg (fibrinogen), CRP (c reactive protein), LVEF (left ventricular ejection fraction).

## DISCUSSION

End-stage renal disease (ESRD) is a significant risk factor for stroke. The risk of all classic stroke sub-types is consistently documented to be increased in CKD and dialysis patients.^1^ However, regardless of the type of stroke, the need for renal replacement therapy in ESRD patients remains consistent. There appear to be hemodialysis relevant risk factors that influence stroke occurrence, such as cerebral hypoperfusion, increased arterial stiffness, and broad blood pressure variation.^2^ Hence, it is crucial for us to be aware that the period of dialysis induction is a high-risk phase for patients who have a high risk for stroke.^3^ This patient developed SH at the early stage of hemodialysis and recovered significantly after conservative treatment.

The differential diagnosis of SH should include ruptured cerebral aneurysm, cerebral vascular malformation, hypertensive cerebral hemorrhage, and traumatic brain injury. In this case, the possibilities of cerebral aneurysm and cerebral vascular malformation were ruled out through cranial MRI and angiography, leading to the final diagnosis of SH.

There are numerous challenges that one must consider when it comes to dialysis in acute brain injury, specifically with regard to stroke. Firstly, it is important to note that intermittent hemodialysis can potentially lead to an increase in brain water content. This increase can, in turn, result in a rise in intracranial pressure. It has been observed that even stable patients can experience subclinical cerebral edema during intermittent hemodialysis. Secondly, osmolality changes during hemodialysis can further complicate the situation. The reverse osmotic shift caused by urea or other newly formed brain osmoles can accentuate intracranial pressure. Additionally, fluctuations in blood pressure and volume have the potential to extend the penumbra in patients with acute stroke. Global cerebral blood flow has been shown to decrease acutely by 10% during hemodialysis, and intradialytic hypotension has been connected to cerebral ischemia. Lastly, it is important to consider the effects of systemic anticoagulation during hemodialysis. Such anticoagulants can potentially contribute to hemorrhage, worsen cerebral edema, and increase the risk of cerebral herniation.

IRR, also known as Wunderlich syndrome, is a rare, but potentially fatal disease.^4^ It is characterized by acute spontaneous renal capsule, perirenal, and postperitoneal hemorrhage and rupture of renal parenchyma and renal pelvis without trauma. If the diagnosis and treatment are not timely, hemorrhagic shock or even life-threatening conditions can occur mostly due to its pathological kidneys.^5^ IRR is a urological emergency that typically has specific underlying causes, with only a few cases remaining unexplained. These causes can include renal tumors, inflammation, stones, vascular conditions, and coagulation disorders, among others. The most straightforward imaging test for diagnosing IRR is an ultrasound, although it may not be as sensitive as CT in determining the nature of fluid accumulation. CT has a diagnostic accuracy of over 90% for the diagnosis of IRR, and it is also superior to ultrasound in the differentiation of solid masses, particularly when using contrast-enhanced CT scans.^6^ Our patient was diagnosed with COVID-19 before admission and suffered from IRR next.

Patients with uremia have abnormal platelet adhesion and aggregation function, leading to a tendency to bleed. Although the primary kidneys disease may vary, both kidneys often experience sclerosis and atrophy. Additionally, renal vascular lesions, hemodynamic changes, and the use of low molecular weight heparin during dialysis can increase the risk of bleeding.^7^ Various spontaneous bleeding complications have been observed following infection with COVID-19, including conditions like retroperitoneal hematoma, gastrointestinal bleeding, hemopneumothorax, and cerebral hemorrhage. Proposed mechanisms for COVID-associated coagulopathy involve the direct infection of endothelial cells through ACE-2 receptors and the production of autoantibodies against endothelial cells, which can develop at a later stage. Prolonged PT, APTT, and INR, as well as elevated levels of D-dimer, have been linked to unfavorable outcomes in COVID-19 patients.^8^ These markers can be considered by emergency medicine physicians who suspect the presence of a COVID-related coagulopathy.

The differential diagnosis of IRR should include renal tumors (such as renal cell carcinoma, angiomyolipoma), renal infections (such as renal abscess), renal stones, renal vascular diseases (such as renal artery aneurysm, pseudoaneurysm), and coagulation disorders. Additionally, traumatic renal injury should also be ruled out. In this case, these possibilities were excluded through CT and angiography, leading to the final diagnosis of spontaneous renal rupture.

The treatment for IRR should prioritize improving symptoms, maintaining vital signs, and preserving kidney function. In addition to conservative treatments such as hemostasis, pain relief, fluid replacement, blood transfusion, and antimicrobial therapy, a personalized plan should be developed based on the location and volume of the ruptured hemorrhage, as well as the underlying kidney disease.^9^ Examples of individualized approaches may include, SRAE, nephrectomy, or partial nephrectomy. Thus, prompt diagnosis and treatment are critical. Angiography as well as, more lately, SRAE are emerging as efficient diagnostic and therapeutic methods for IRR.^10^ This patient was detected using CT in the situation of active bleeding and SRAE was performed successfully for him. Hence, SRAE was safely and effectively performed for our patient and he recovered quickly after surgery.

Additionally, uremia patients who suffer from IRR and SH have the potential to imitate other medical conditions, and this can ultimately result in life-threatening hemorrhagic shock. Given the knowledge that COVID-19 infection can heighten the risk of bleeding, it is crucial for medical practitioners to maintain a state of heightened awareness regarding the occurrence of spontaneous events. When it comes to emergency treatment, it is important to consider actions such as transfusing blood products, reversing the effects of anticoagulant medications, and seeking prompt consultation from surgical, critical care, and interventional radiology experts.

### Limitations:

The limitations of this case include: 1) This is a single case report, lacking support from large sample data; 2) The patient’s COVID-19 infection may have a potential impact on the occurrence of IRR and SH, but due to the limited number of cases, a clear causal relationship cannot be established; 3) The long-term follow-up data of the patient is insufficient, making it difficult to assess the long-term prognosis.

## CONCLUSION

To the best of our knowledge, this is the first reported case of a hemodialysis patient with COVID-19 who suffered from SH concomitant with IRR. Hence, SH and IRR should be considered in hemodialysis patients with COVID-19 who have a sudden headache or abdominal pain. CT scan should be performed as soon as possible for a clear diagnosis and timely treatment is necessary accordingly.
